# Reduction Formulae for Products of Theta Functions

**DOI:** 10.6028/jres.117.017

**Published:** 2012-09-06

**Authors:** P. L. Walker

**Affiliations:** 7 Redgrove Park, Cheltenham, GL51 6QY UK

**Keywords:** Jacobian theta functions, especially products, normalizing factors

## Abstract

In four cases it is already known that the product of two distinct Jacobian theta functions having the same variable *z* and the same nome *q* is a multiple of a single Jacobian theta function, with the multiple independent of *z*. The main purpose of the present note is to show that this property also applies in the remaining two cases.

## 1. Introduction

The formulae

(1) $$\frac{\theta_{1}(z,q)\theta_{2}(z,q)}{\theta_{1}(2z,q^{2})}=\frac{\theta_{3}(z,q)\theta_{4}(z,q)}{\theta_{4}(2z,q^{2})}=\theta_{4}(0,q^{2}),$$

(2) $$\frac{\theta_{1}(z,q^{2})\theta_{4}(z,q^{2})}{\theta_{1}(z,q)}=\frac{\theta_{2}(z,q^{2})\theta_{3}(z,q^{2})}{\theta_{2}(z,q)}=\frac{1}{2}\theta_{2}(0,q),$$

occur in the theory of Jacobian theta functions and their applications to Jacobian elliptic functions. For example, Eq. (1) leads to the Landen transformations (Ref. [1], Subsection 20.7(vi) and Section 22.7).

The main purpose of the present paper is to prove that

(3) $$\frac{\theta_{1}(z,q^{2})\theta_{3}(z,q^{2})}{\theta_{1}(z,iq)}=\frac{\theta_{2}(z,q^{2})\theta_{4}(z,q^{2})}{\theta_{2}(z,iq)}=i^{-1/4}\sqrt{\frac{\theta_{2}(0,q^{2})\theta_{4}(0,q^{2})}{2},}$$

thereby covering the remaining two cases.

However, we shall also prove Eqs. (1) and (2) by the same method, thereby providing new proofs for these equations. Incidentally, since the right-hand sides of Eqs. (1), (2), and (3) are independent of *z*, we shall refer to them as *normalizing factors*.

## 2. Infinite Products

We begin with the well-known expansions of the theta functions as infinite products (Ref. [1], Eqs.(20.5.1)-(20.5.4)):

(4) $$\begin{array}{l} \theta_{1}(z,q)=2q^{1/4}\sin z\prod_{n=1}^{\infty }(1-q^{2n})(1-2q^{2n}\cos(2z)+q^{4n}),\\ \theta_{2}(z,q)=2q^{1/4}\cos z\prod_{n=1}^{\infty }(1-q^{2n})(1+2q^{2n}\cos(2z)+q^{4n}),\\ \theta_{3}(z,q)=\prod_{n=1}^{\infty }(1-q^{2n})(1+2q^{2n-1}\cos(2z)+q^{4n-2}),\\ \theta_{4}(z,q)=\prod_{n=1}^{\infty }(1-q^{2n})(1-2q^{2n-1}\cos(2z)+q^{4n-2}). \end{array}$$

In particular,

(5) $$\begin{array}{l} {\theta^{\prime }}_{1}(0,q)=2q^{1/4}\prod_{n=1}^{\infty }{\left(1-q^{2n}\right)}^{3},\\ \theta_{2}(0,q)=2q^{1/4}\prod_{n=1}^{\infty }\left(1-q^{2n}\right){\left(1+q^{2n}\right)}^{2},\\ \theta_{3}(0,q)=\prod_{n=1}^{\infty }\left(1-q^{2n}\right){\left(1+q^{2n-1}\right)}^{2},\\ \theta_{4}(0,q)=\prod_{n=1}^{\infty }\left(1-q^{2n}\right){\left(1-q^{2n-1}\right)}^{2}. \end{array}$$

The prime in ${\theta^{\prime }}_{1}(0,q)$ indicates differentiation with respect to the variable *z*. In these formulae, *z*,*q* are complex and |*q*| < 1 to ensure convergence. Here *q*^1/4^ denotes the principal value of the root. With *q* = *e^iπτ^*, *τ* is a complex parameter with positive imaginary part.

With this notation we have

(6) $$\begin{array}{l} 1-2q^{2n}\cos(2z)+q^{4n}=1-2e^{2ni\pi \tau }\cos(2z)+e^{4ni\pi \tau }\\ \;=2e^{2ni\pi \tau }(\cos(2n\pi \tau )-\cos(2z))\\ \;=4e^{2ni\pi \tau }\sin(z-n\pi \tau )\sin(z+n\pi \tau ), \end{array}$$

and hence

(7) $$\frac{\theta_{1}(z,q)}{{\theta^{\prime }}_{1}(0,q)}=\sin z\prod_{n=1}^{\infty }\frac{\sin(n\pi \tau -z)\sin(n\pi \tau +z)}{\sin^{2}(n\pi \tau )}$$

Note that as *^n^* → ∞, the *n* th factor in this infinite product is 1+ *O*(*e*^2^*^niπτ^*), which ensures rapid convergence since ℑ*τ* > 0

Repetition of this calculation (details omitted) for *θ*_2_, *θ*_3_, and *θ*_4_ yields

(8) $$\frac{\theta_{2}(z,q)}{\theta_{2}(0,q)}=\cos z\prod_{n=1}^{\infty }\frac{\cos(n\pi \tau -z)\cos(n\pi \tau +z)}{\cos^{2}(n\pi \tau )},$$

(9) $$\frac{\theta_{3}(z,q)}{\theta_{3}(0,q)}=\prod_{n=1}^{\infty }\frac{\cos\left(\left(n-\frac{1}{2}\right)\pi \tau -z\right)\cos\left(\left(n-\frac{1}{2}\right)\pi \tau +z\right)}{\cos^{2}\left(\left(n-\frac{1}{2}\right)\pi \tau \right)}$$

and

(10) $$\frac{\theta_{4}(z,q)}{\theta_{4}(0,q)}=\prod_{n=1}^{\infty }\frac{\sin\left(\left(n-\frac{1}{2}\right)\pi \tau -z\right)\sin\left(\left(n-\frac{1}{2}\right)\pi \tau +z\right)}{\sin^{2}\left(\left(n-\frac{1}{2}\right)\pi \tau \right)}.$$

The required results now follow on combining these equations in pairs.

For the product *θ*_1_ *θ*_2_ we have from Eqs. (7) and (8)

(11) $$\begin{array}{c} \frac{\theta_{1}(z,q)\theta_{2}(z,q)}{{\theta^{\prime }}_{1}(0,q)\theta_{2}(0,q)}=\sin z\cos z\prod_{n=1}^{\infty }\frac{\sin(n\pi \tau -z)\sin(n\pi \tau +z)}{\sin^{2}(n\pi \tau )}\\ \times \frac{\cos(n\pi \tau -z)\cos(n\pi \tau +z)}{\cos^{2}(n\pi \tau )}\\ \;=\frac{1}{2}\sin(2z)\prod_{n=1}^{\infty }\frac{\sin(2n\pi \tau -2z)\sin(2n\pi \tau +2z)}{\sin^{2}(2n\pi \tau )}\\ =\frac{1}{2}\frac{\theta_{1}(2z,q^{2})}{{\theta^{\prime }}_{1}(0,q^{2})}, \end{array}$$

where we have used the identity *q*^2^ = *e*^2^*^iπτ^*. Hence

(12) $$\frac{\theta_{1}(z,q)\theta_{2}(z,q)}{\theta_{1}(2z,q^{2})}=\frac{1}{2}\frac{{\theta^{\prime }}_{1}(0,q)\theta_{2}(0,q)}{{\theta^{\prime }}_{1}(0,q^{2})}$$

The translation $z\to z+\frac{1}{2}\tau$ changes *θ*_1_→*θ*_3_ and *θ*_2_→*θ*4. Hence we have

(13) $$\frac{\theta_{1}(z,q)\theta_{2}(z,q)}{\theta_{1}(2z,q^{2})}=\frac{\theta_{3}(z,q)\theta_{4}(z,q)}{\theta_{3}(2z,q^{2})}=\frac{1}{2}\frac{{\theta^{\prime }}_{1}(0,q)\theta_{2}(0,q)}{{\theta^{\prime }}_{1}(0,q^{2})},$$

which is the first required result, Eq. (1), except for the normalizing factor. We will return to this below and for now concentrate on the quotients of functions of *z*.

Turning to the product*θ*_1_ *θ*_4_, we have from Eqs. (7) and (10)

(14) $$\begin{array}{l} \frac{\theta_{1}(z,q^{2})\theta_{4}(z,q^{2})}{{\theta^{\prime }}_{1}(0,q^{2})\theta_{4}(0,q^{2})}=\sin z\prod_{n=1}^{\infty }\frac{\sin(2n\pi \tau -z)\sin(2n\pi \tau +z)}{\sin^{2}(2n\pi \tau )}\\ \;\times \frac{\sin\left(\left(2n-1\right)\pi \tau -z\right)\sin((2n-1)\pi \tau +z)}{\sin^{2}((2n-1)\pi \tau )}\\ \;=\sin z\prod_{n=1}^{\infty }\frac{\sin(n\pi \tau -z)\sin(n\pi \tau +z)}{\sin^{2}(n\pi \tau )}=\frac{\theta_{1}(z,q)}{{\theta^{\prime }}_{1}(0,q)}, \end{array}$$

where in the last line we have combined the even(2n)and the odd (2*n* −1) terms to obtain the complete sequence. Hence on rearranging we have

(15) $$\frac{\theta_{1}(z,q^{2})\theta_{4}(z,q^{2})}{\theta_{1}(z,q)}=\frac{{\theta^{\prime }}_{1}(0,q^{2})\theta_{4}(0,q^{2})}{{\theta^{\prime }}_{1}(0,q)},$$

followed by translation in by z by $\frac{1}{2}\pi :$

(16) $$\frac{\theta_{1}(z,q^{2})\theta_{4}(z,q^{2})}{\theta_{1}(z,q)}=\frac{\theta_{2}(z,q^{2})\theta_{3}(z,q^{2})}{\theta_{2}(z,q)}=\frac{{\theta^{\prime }}_{1}(0,q^{2})\theta_{4}(0,q^{2})}{{\theta^{\prime }}_{1}(0,q)},$$

which is Eq. (2), except for the normalizing factor.

For the third identity we note that *q* → *iq* corresponds to $\tau \to \tau +\frac{1}{2}$. From Eqs. (7) and (9) we have

(17) $$\begin{array}{l} \frac{\theta_{1}(z,q^{2})\theta_{3}(z,q^{2})}{{\theta^{\prime }}_{1}(0,q^{2})\theta_{3}(0,q^{2})}=\sin z\prod_{n=1}^{\infty }\frac{\sin(2n\pi \tau -z)\sin(2n\pi \tau +z)}{\sin^{2}(2n\pi \tau )}\\ \;\times \frac{\cos((2n-1)\pi \tau -z)\cos((2n-1)\pi \tau +z)}{\cos^{2}((2n-1)\pi \tau )}, \end{array}$$

and

(18) $$\frac{\theta_{1}(z,iq)}{{\theta^{\prime }}_{1}(0,iq)}=\sin z\prod_{n=1}^{\infty }\frac{\sin\left(n\pi \left(\tau +\frac{1}{2}\right)-z\right)\sin\left(n\pi \left(\tau +\frac{1}{2}\right)+z\right)}{\sin^{2}\left(n\pi \left(\tau +\frac{1}{2}\right)\right)}.$$

Now in Eq. (18), we separate the terms according to the parity of *n.*

If is even, then *n* = 2*k* (*k* ≥ 1) is even then

(19) $$\sin(n\pi (\tau +\frac{1}{2})\pm z)={\left(-1\right)}^{k}\sin(2k\pi \tau \pm z),$$

and

(20) $$\sin^{2}(n\pi (\tau +\frac{1}{2}))=\sin^{2}(2k\pi \tau ),$$

corresponding to the first quotient on the right-hand side of Eq. (17).

Similarly if *n* = 2*k* −1 (*k* ≥ 1) is odd, then

(21) $$\begin{array}{l} \sin(n\pi (\tau +\frac{1}{2})\pm z)=\sin((2k-1)\pi (\tau +\frac{1}{2})\pm z)\\ \;={\left(-1\right)}^{k}\cos((2k-1)\pi \tau \pm z), \end{array}$$

and

(22) $$\sin^{2}((2k-1)\pi (\tau +\frac{1}{2}))=\cos^{2}((2k-1)\pi \tau ),$$

corresponding to the second quotient in Eq. (17). Hence

(23) $$\frac{\theta_{1}(z,q^{2})\theta_{3}(z,q^{2})}{{\theta^{\prime }}_{1}(0,q^{2})\theta_{3}(0,q^{2})}=\frac{\theta_{1}(z,iq)}{{\theta^{\prime }}_{1}(0,iq)},$$

or

(24) $$\frac{\theta_{1}(z,q^{2})\theta_{3}(z,q^{2})}{\theta_{1}(z,iq)}=\frac{{\theta^{\prime }}_{1}(0,q^{2})\theta_{3}(0,q^{2})}{{\theta^{\prime }}_{1}(0,iq)},$$

and translation in *z* by $\frac{1}{2}\pi$gives

(25) $$\frac{\theta_{1}(z,q^{2})\theta_{3}(z,q^{2})}{\theta_{1}(z,iq)}=\frac{\theta_{2}(z,q^{2})\theta_{4}(z,q^{2})}{\theta_{2}(z,iq)}=\frac{{\theta^{\prime }}_{1}(0,q^{2})\theta_{3}(0,q^{2})}{{\theta^{\prime }}_{1}(0,iq)},$$

which is Eq. (3) apart from the normalizing factor.

## 3. Normalizing Factors

If we compare our results (13), (16), and (25), with the desired Eqs. (1), (2), and (3), then we see that we have to verify the following identities:

(26) $$\frac{1}{2}\frac{{\theta^{\prime }}_{1}(0,q)\theta_{2}(0,q)}{{\theta^{\prime }}_{1}(0,q^{2})}=\theta_{4}(0,q^{2}),$$

(27) $$\frac{{\theta^{\prime }}_{1}(0,q^{2})\theta_{4}(0,q^{2})}{{\theta^{\prime }}_{1}(0,q)}=\frac{1}{2}\theta_{2}(0,q),$$

and

(28) $$\frac{{\theta^{\prime }}_{1}(0,q^{2})\theta_{3}(0,q^{2})}{{\theta^{\prime }}_{1}(0,iq)}=i^{-1/4}\sqrt{\frac{\theta_{2}(0,q^{2})\theta_{4}(0,q^{2})}{2}}.$$

The following identity, which goes back at least to Euler, is needed. When | *q* |< 1

(29) $$\begin{array}{l} P(q)=\prod_{n=1}^{\infty }\left(1+q^{n})(1-q^{2n-1}\right)\\ \;=\{(1+q)(1-q)\}\{(1+q^{2})(1-q^{3})\}\{(1+q^{3})(1-q^{5})\}\cdots =1. \end{array}$$

To verify this, we note that on expanding the product in powers of *q*, only a finite number of factors are needed to determine the coefficient of each power, so that no limiting process is involved and the terms may be re-ordered without affecting the value of the product. (Alternatively, for an analytic argument, the rearrangement is justified by absolute convergence.) Therefore

(30) $$\begin{array}{l} P(q)=\{(1+q)(1+q^{2})(1-q)\}\{(1+q^{3})(1+q^{4})(1-q^{3})\}\\ \;\times \{(1+q^{5})(1+q^{6})(1-q^{5})\}\cdots \\ \;=\{(1+q^{2})(1-q^{2})\}\{(1+q^{4})(1-q^{6})\}\{(1+q^{6})(1-q^{10})\}\cdots \\ \;=\prod_{n=1}^{\infty }\{(1+q^{2n})(1-q^{4n-2})\}=P(q^{2}). \end{array}$$

If *P*(*q*)is not identically 1, then there will be a least power of *q*, say *q^k^*, whose coefficient is not zero. But then *q*^2^*^k^* will be the least power of *q* in *P*(*q*^2^) whose coefficient is not zero, so *P*(*q*) and *P*(*q*^2^) cannot be equal, resulting in a contradiction.

To prove Eq. (26) we substitute from Eqs. (5), and see that we require

(31) $${\left(2q^{1/4}\right)}^{2}\prod_{n=1}^{\infty }{\left(1-q^{2n}\right)}^{4}{\left(1+q^{2n}\right)}^{2}=2(2q^{1/2})\prod_{n=1}^{\infty }{\left(1-q^{4n}\right)}^{4}{\left(1-q^{4n-2}\right)}^{2}.$$

After division by $4q^{1/2}\prod_{n=1}^{\infty }{\left(1-q^{2n}\right)}^{2}(1+q^{2n})$, we see that the last equation is equivalent to

(32) $$\prod_{n=1}^{\infty }{\left(1-q^{2n}\right)}^{2}\left(1+q^{2n}\right)=\prod_{n=1}^{\infty }{\left(1-q^{4n}\right)}^{2}(1-q^{4n-2}).$$

Replacement of the term (1− *q*^4^*^n^*) by (1− *q*^2^*^n^*)(1+ *q*^2^*^n^*), and use of Eq. (29) with *q*^2^ in place of *q*, completes the proof.

For Eq. (27) we require similarly

(33) $$2q^{1/2}\prod_{n=1}^{\infty }{\left(1-q^{4n}\right)}^{4}{\left(1-q^{4n-2}\right)}^{2}=\frac{{\left(2q^{1/4}\right)}^{2}}{2}\prod_{n=1}^{\infty }{\left(1-q^{2n}\right)}^{4}{\left(1+q^{2n}\right)}^{2},$$

or, on division by $2q^{1/2}\prod_{n=1}^{\infty }{\left(1-q^{4n}\right)}^{2}\left(1-q^{4n-2}\right):$

(34) $$\prod_{n=1}^{\infty }{\left(1-q^{4n}\right)}^{2}\left(1-q^{4n-2}\right)=\prod_{n=1}^{\infty }{\left(1-q^{2n}\right)}^{2}\left(1+q^{2n}\right).$$

This completes the proof of Eq. (27).

Finally, for Eq. (28) we begin by calculating the asymptotic values of each side as through *q* → 0 through positive real values. On the left-hand side we have

(35) $$\left\{2{\left(q^{2}\right)}^{1/4}/\left(2{\left(iq\right)}^{1/4}\right)\right\}\left(1+O\left(q^{2}\right)\right)={\left(q/i\right)}^{1/4}\left(1+O\left(q^{2}\right)\right).$$

On the right-hand side we have $i^{-1/4}\sqrt{2{\left(q^{2}\right)}^{1/4}/2\left(1+O\left(q^{2}\right)\right)}$, which is the same. Hence we can simply square both sides and compare the results. For the left-hand side we have

(36) $$\begin{array}{l} {\left(\frac{{\theta^{\prime }}_{1}\left(0,q^{2}\right)\theta_{3}\left(0,q^{2}\right)}{{\theta^{\prime }}_{1}\left(0,iq\right)}\right)}^{2}=\frac{{\left(q^{2}\right)}^{1/2}}{{\left(iq\right)}^{1/2}}\prod_{n=1}^{\infty }\frac{{\left(1-q^{4n}\right)}^{8}}{{\left(1-{\left(-1\right)}^{n}q^{2n}\right)}^{6}}{\left(1+q^{4n-2}\right)}^{4}\\ \;={\left(\frac{q}{i}\right)}^{1/2}\prod_{n=1}^{\infty }\frac{{\left(1-q^{4n}\right)}^{2}}{{\left(1+q^{4n-2}\right)}^{2}}. \end{array}$$

For the right-hand side we have

(37) $$i^{-1/2}\frac{\theta_{2}\left(0,q^{2}\right)\theta_{4}\left(0,q^{2}\right)}{2}=\frac{2i^{-1/2}q^{1/2}}{2}\prod_{n=1}^{\infty }{\left(1-q^{4n}\right)}^{2}{\left(1+q^{4n}\right)}^{2}{\left(1-q^{4n-2}\right)}^{2}.$$

On comparison of the two sides we see that the result to be proved is given by

(38) $$1=\prod_{n=1}^{\infty }{\left(1+q^{4n-2}\right)}^{2}{\left(1+q^{4n}\right)}^{2}{\left(1-q^{4n-2}\right)}^{2},$$

or equivalently,

(39) $$1=\prod_{n=1}^{\infty }\left(1+q^{4n}\right)\left(1-q^{8n-4}\right),$$

which is Eq. (29) with *q*^4^ in place of *q*.
